# Four-Day-Old Human Neonates Look Longer at Non-Biological Motions of a Single Point-of-Light

**DOI:** 10.1371/journal.pone.0000186

**Published:** 2007-01-31

**Authors:** David Méary, Elenitsa Kitromilides, Karine Mazens, Christian Graff, Edouard Gentaz

**Affiliations:** Psychology and NeuroCognition Laboratory, Centre National de la Recherche Scientifique (CNRS), UMR 5105, University Pierre Mendès France, Grenoble, France; James Cook University, Australia

## Abstract

**Background:**

Biological motions, that is, the movements of humans and other vertebrates, are characterized by dynamic regularities that reflect the structure and the control schemes of the musculo-skeletal system. Early studies on the development of the visual perception of biological motion showed that infants after three months of age distinguished between biological and non-biological locomotion.

**Methodology/Principal Findings:**

Using single point-light motions that varied with respect to the “two-third-power law” of motion generation and perception, we observed that four-day-old human neonates looked longer at non-biological motions than at biological motions when these were simultaneously presented in a standard preferential looking paradigm.

**Conclusion/Significance:**

This result can be interpreted within the “violation of expectation” framework and can indicate that neonates' motion perception — like adults'—is attuned to biological kinematics.

## Introduction

The movements of humans and other vertebrates, (i.e., biological motions) form a class of particularly salient visual stimuli [1]–[3]. This was first observed by Johansson with his famous “point-light” paradigm [1]. He attached small lights to the joints of actors and filmed them executing different activities. The final movies showed a dozen of moving point-of-lights only, but adult participants reported a vivid and compelling experience of a person walking, dancing, or cycling. No recognition occurred when single frames (i.e., static images) were shown. Despite the apparent complexity of locomotion its recognition took about 200 ms only [2], suggesting that the analysis of biological motion could be an intrinsic feature of human's visual system [1]–[3], and may depend on core knowledge [4].

The origin and the development of biological motion perception in humans were first studied by Fox and McDaniel [5]. They showed that infants from 4-months of age preferred an upright point-light walker to the same pattern turned upside-down. A visual preference for biological locomotion was confirmed in 3- and 5-month-old infants [6]–[7] but was not found in 2-month-old infants [5], suggesting that the perception of locomotion in point-light displays required some visual experience and/or maturation of visual structures. Besides, it was unclear whether infants failed in extracting the relative motion of the points or whether this relative motion pattern was not yet attractive.

Biological motion effects are not limited to the recognition of displays involving multiple point-of-lights: Motions of a single point-of-light are also often related to the way humans move [3], [8]–[9]. Amongst the most popular laws of natural motion the, so-called, “two-third-power law” relates the curvature and the tangential velocity in planar arm movements [10] as well as in smooth pursuit eye movements [11]. It was shown that 2-D point-light motions that follow this motor principle are experienced as having a uniform speed even though the point-light velocity may vary significantly along the motion path. This is the case for point-light motion along elliptical paths. According to the two-third-power law, the tangential velocity can be tripled between points of maximum and minimum path curvature, and yet appear constant. Conversely, point-light motions with constant tangential velocity along elliptical paths are perceived as having a non-uniform velocity [9]. This illusion of uniform velocity has been discussed within the motor theories of perception which holds that perceptual systems draw on implicit motor knowledge to process biological events [3], [12]. Later studies have proposed instead a purely visual interpretation [13].

The sensitivity to these biological and non-biological motions of a single point-of-light has been investigated in adults only and it is not known how early these two categories of motions are differentiated. We tested 4-day-old human neonates using a standard preferential-looking paradigm [5], [14]. Biological *vs*. non-biological motions of a single point-light were presented simultaneously on two screens. We hypothesized that if the two categories of motion are discernable at birth the neonates' looking behavior will reveal significant contrasts between them.

## Methods

### Participants

Each neonate was tested once for the visual preference between two motions of a single point-of-light. The sample consisted of 84 full-term neonates (38 girls and 46 boys) from the maternity home of the “Clinique Mutualiste” in Grenoble (France). Their mean age was about 101 hr (min 50 hr, max 130 hr). Neonates were tested in the morning, at the hospital nursery, just before or after standard medical examinations. All the tested neonates were judged in good health, with normal weight, and in a receptive mood by a pediatrician. Only one out of three neonates viewed in the morning was judged receptive enough, at this time, to participate to the study.

### Materials

Each neonate was positioned by its caregiver in an adapted rigid seat, fixed on a trolley, covered with a blanket and inclined by 30°. Two adjustable cushions were placed on both sides of the neonate's head, increasing head inclination by 10°. Just before the experiment started, we positioned the trolley in front of the visual display. The display consisted of two identical LCD screens (1280×1024 pixels, 37.6×30.2 cm), separated by a 5 cm gap. The distance between the neonate's eyes and the screens was 35–40 centimeters. A digital video camera, placed between the two screens, recorded the neonate's behavior during the test phase at a rate of 25 frames per second (fps). The screens' borders, the camera, and the experimenter controlling the apparatus were hidden from the neonate's sight by a large black cardboard.

### Stimuli and Methods

**Figure 1:**
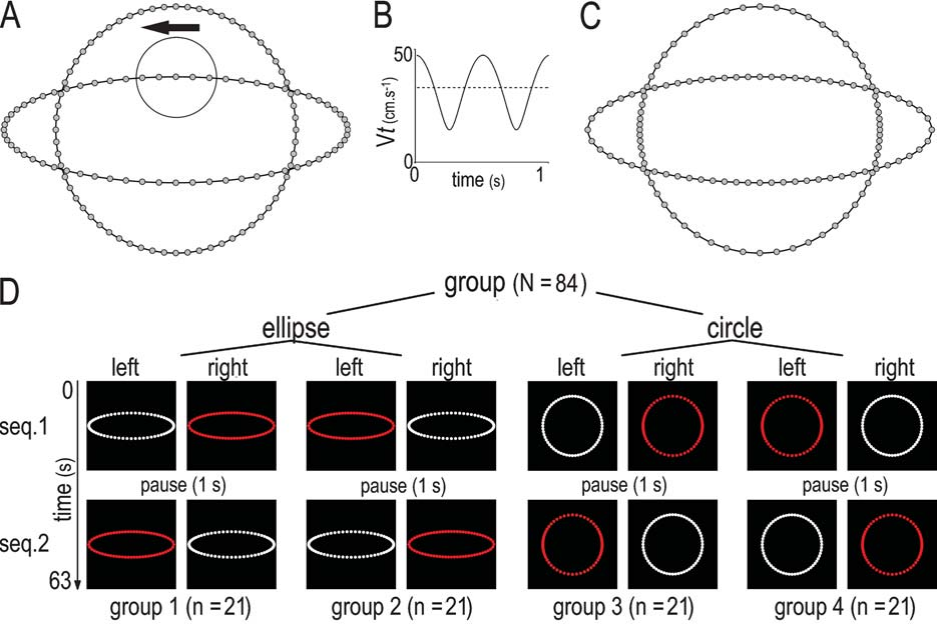
Stimuli and Experimental Design. (A) Geometry (line) and Cartesian coordinates (markers) of the biological stimuli. The arrow indicates the direction of motion. The circular outline shows the starting position and the relative size of the light-spot with respect to the trajectory. (B) Tangential velocity of the elliptical (line) and circular (dashed line) biological stimuli. (C) Non-biological motions derived from the biological coordinates. Geometry and kinematics were inverted. (D) Experimental design. The neonates were assigned to one of the four sub-groups defined by the combination of the stimulus geometry (ellipse or circle) and of the screen (left or right) displaying the biological motion (white points)

The single dot motions displayed on the screens resulted from the combination of two shapes and two laws of motion (Figure 1, A–C). First, we defined 65 linearly spaced values for an angle *θ* (from *π*/2 by step of −*π*/32). The *x* and *y* coordinates for the elliptical and the circular biological motion were given by *x* = *a*.cos(*θ*) and *y* = *b*.sin(*θ*), with *a* = 1 and *b* = 0.3 in the case of the ellipse, and *a*′ = *b*′ = 0.698 in the case of the circle (giving equivalent path length). The laws of motion *s* = *s*(*t*) corresponding to these coordinates conformed to the 2/3 power law and could be considered as instances of biological motions [12]. Then, we recombined the two laws of motion and the two shapes to build two non-biological sets of coordinates (Video S1 and Video S2). In practice, we used a polar definition of the shapes and computed the 65 values of the angle *θ* satisfying the law of motion of the ellipse along a circle, and vice versa. We recall that for a given path shape there is only one biological motion (the one satisfying the two-third-power law) but many non-biological motions (which are not equally discernable). Our choice guaranteed that the biological motion could not be associated to a unique path shape nor to a unique law of motion.

Using the 4 sets of coordinates, we built movies with spatial and temporal resolution of 640 by 512 pixels and 60 fps respectively. When displayed full screen, the light-spot (220 candelas [cd]/m^2^, Ø = 110 pixels) traveled on a dark background (0.6 cd/m^2^) along a 37 cm circular or elliptical path with an average tangential velocity of 34.6 cm.s^−1^. One sequence comprised 31 s of uninterrupted motion (29 cycles) with no appreciable flickering. Each test included two sequences separated by a 1 s black screen. During the test phase, the two movies showing a circular motion (or those showing an elliptical motion) were played simultaneously on the screens. We counterbalanced the position of the biological stimuli, both within and between the neonates, to control possible bias related to the screen (see Figure 1, D).

### Statistical Analysis

Records of the neonate's behavior during the test were analyzed off-line, on a frame by frame basis, by an independent judge unaware of the experimental conditions. He had to classify the neonate's behavior according to three categories: gaze directed to the right screen, gaze directed to the left screen, or gaze directed elsewhere (including eyes-blinking, sneezing, yawning, etc.). The judge eliminated 11 neonates from the sample because he was unable to estimate gaze direction due to a poor positioning of the neonates' head or a poor camera focus. Finally, knowing the neonate's group and the sequence, we expressed the looking behavior of the 73 remaining neonates with respect to the displayed motions (biological vs. non-biological). Two criteria were established for including the data from an individual neonate: A maximum of 50% of the stimulus duration spent elsewhere, and a minimum of 15% of the remaining time spent looking at each screen. Seven neonates did not meet the maximum elsewhere criterion and 15 neonates did not meet the minimum looking criterion (they focused on one of the two screens). These criteria guaranteed that each neonate looked at each screen for about 5 s at least (i.e., about 4 cycles of the motion). The final sample comprised 51 neonates (ellipse group: n = 27; circle group: n = 24). Analysis of variance (ANOVA) of the mean looking time was used to assess the effect of the type of motion. The path shape and the sequence were considered as between and within-subjects independent variables, respectively.

## Results

Eighty-four neonates underwent a 63-seconds free-choice test involving two different animations in a standard preferential-looking paradigm. The means of the looking time for each stimulus and within each sequence are presented in Table 1. The contributions of the type of motion, the sequence, and the path shape to the mean looking time were assessed using ANOVA. The type of motion and the sequence were treated as within-subjects factors and the path shape was treated as a between-subjects factor. The type of motion and the sequence influenced the mean looking time (type of motion: *F*_(1,49)_ = 4.97, *p* = .03; sequence: *F*_(1,49)_ = 5.55, *p* = .022) but the effect of the path shape was not significant (*F*_(1,49)_ = 0.23, *p* = .63). To resume these results: the neonates spent more time looking at the non-biological motions and they spent less time looking at the motions during the second sequence. The proportion of the total variance explained by the type of motion and the sequence was 4% and 0.5% respectively, given by the η^2^ measure of effect size that is the sum of square (SS) of the effect/(SS of the effects + SS of the errors). All the interactions between type of motion, sequence, and path shape were not significant. Finally, the small effect of the type of motion seemed to depend on the sequence although the interaction was not significant. Planned comparison confirmed that the type of motion was mostly influential during the first sequence (sequence 1: *F*_(1,49)_ = 4.27, *p* = .044; sequence 2: *F*_(1,49)_ = 1.05, *p* = .30).

**Table 1:** Means of the looking time (LT) as a function of the type of motion (biological, B; non-biological, NB), the path shape, and the sequence.

	Looking Time (s)		Total	LT in proportion	
Path	B	NB	T = B+NB	B	NB
	*Sequence 1*				
Elliptic (n = 27)	10.81	13.65	24.46	0.44	0.56
Circular (n = 24)	9.97	14.25	24.22	0.41	0.59
Mean 1	10.39	13.95*	24.34	0.43	0.57
	*Sequence 2*				
Elliptic	10.43	12.35	22.78	0.46	0.54
Circular	9.87	11.96	21.83	0.45	0.55
Mean 2	10.15	12.16	22.31	0.45	0.55
Total (N = 51)	20.54	26.11*	46.65	0.44	0.56
IC 95%	2.40	2.98		0.05	

Asterisks indicate significant differences between the biological and the non-biological motion (* *p*< = .05).

**Figure 2:**
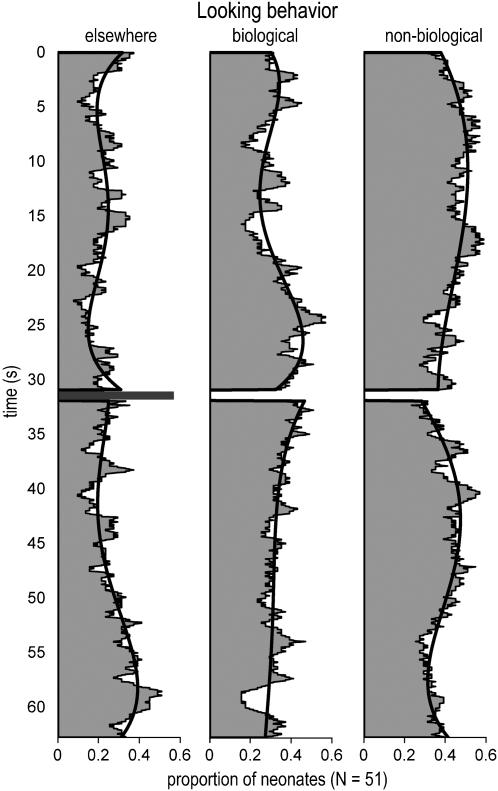
Frequency of the three behaviors in the neonates sample as a function of time. It provides an estimate of the time-varying probability of observing a given behavior. The gray bar corresponds to the 1 s pause. The data were fitted with a fourth order polynomial to figure out the global trend.

This general analysis, showing a main effect of the type of motion on the mean looking time during the first sequence only, suggested that the neonates' behavior changed over time. This is not surprising given that habituation may occur during the 63 s of the test. Figure 2 illustrates the average behavior of the sample of neonates over time. During an initial period of 5 s, the proportion of neonates looking elsewhere decreased suggesting that the moving stimuli retained their interest. This proportion subsequently stabilized between 0.15 and 0.30 and remained roughly constant over a period of 40 s but increased near the end of the testing time.

From the general analysis, we know that the two path shapes (circle and ellipse) led to fairly similar data thus we pooled the two groups and focused on the distinction between the biological and the non-biological type of motion. During the first seconds of the test, the proportion of neonates observing the biological motions and the proportion of neonates observing the non-biological motions were roughly equivalent, at about 0.35. Following this initial period of indifference, however, the proportion of neonates looking at the non-biological motions increased, suggesting that these were more attractive at this time. Following the pause (1 s of black screens), the presentation sides of the biological and non-biological motions were inverted. The neonates who were observing a non-biological motion at the end of the first sequence were thus presented with a biological motion along the same path. Group behavior was very similar during the second sequence. The proportion of neonates looking at the non-biological motions increased and reached a peak at about 40 s (i.e., 8 s after sequence onset). However, as revealed by the general analysis the overall preference for the non-biological motions weakened somehow during this second sequence. Habituation and reduced vigilance could be amongst the main causes of these changes in the proportions of neonates looking at the non-biological motion.

## Discussion

Eye movements in response to a moving object can be observed from the very first day of life [15] and moving stimuli are good at eliciting neonates' interest [16]. Here, we found that changes in the laws of motion of a single point-of-light, with reference to the biological model given in the two-third-power law, led to asymmetries in looking behavior. The looking time was initially increased if the motion of the observed stimulus infringed this hallmark of human movements.

The reason why the neonates looked longer at the non-biological motion requires additional explanations. Longer looking time in preferential looking experiment can have different interpretations. First, the non-biological motion may have violated prior expectations about the dynamics of physical events. Similarly, infants respond to slight changes in the dynamics of a rolling ball [17]–[18] by the age of 7-and 8-months and at 2–3-month-old they already look longer at impossible events in which physical constraints are contravened [4], [19]. At a time interpreted as a transitive preference due to the habituation procedure preceding the test phase [20], violations of expectations were also found in experiments without habituation [21]. Alternatively, we may suppose that the non-biological stimuli were simply more difficult to perceive. In adults, smooth-pursuit eye movements are perturbed if the target motion is at odds with the two-third-power law [11]. Although neonates use mostly fixations and saccades, this problem of dynamic compatibility between the target and the neonate's occulomotor system may also have played a role in this experiment.

What is needed to detect the difference between our biological and non-biological motions? Traditionally, the two-third-power law has been associated with studies of motor-perceptual interactions proposing that implicit motor knowledge could be used by the visual system to interpret dynamic events [3], [12]. Many psychophysical, neurophysiological and neuroimaging studies showed indeed that the humans motor system contributed to the observation, the recognition and the understanding of actions [22]–[24]. However, the single point-light displays used in our experiment can hardly be thought of as a human action and the reduced motor experience of human neonates may be insufficient to permit motor-perceptual mapping. Purely visual interpretations of the two-third-power law suggest instead that the differentiation between our biological and non-biological motions could occur “downstream”. Pollick and Sapiro [13] proposed that the visual system may use affine, rather than Euclidean, geometry, when representing the environment. In line with this view, the two-third-power law of motion perception would reflect an affine perceptual encoding (i.e., a reference frame in which motions are invariant under translation and rotations). Finally, and according to neurophysiological studies of movement production in monkeys the two-third-power law could also be related to the neuron population coding for movement direction [25]. Recent studies on vision in human and non human primates [26]–[29] does not preclude the possibility that the direction-sensitive neural populations involved in motion perception obey the same constraint, in particular at the level of the superior colliculus and of the medial temporal area (MT/V5). Yet, the small size of the effect observed in this study is consistent with the general view that human visual system remains largely immature at birth.

### Conclusion

Given the immaturity of the neonate visual system, an explanation of the perceptual side of the two-third power law in terms of neurofunctional constraints at the level of motion-sensitive areas seems more parsimonious than the motor-perceptual hypothesis but the question is not answered yet. At least, our experimental results indicate that the looking behavior of 4-day-old human neonates may already be influenced by motions that contravene the two-third-power law.

### Ethical considerations

This experiment is part of a larger project focusing on the perceptual abilities of human neonates. All the experiments were approved by a committee of pediatricians, nurses, and parents from the maternity home of the “clinique mutualiste” in Grenoble. A committee from the French National Center for Scientific Research also approved the project. This experiment has been classified as purely behavioral testing involving no distress or discomfort to the neonates at all. At least one of the neonates' parents gave informed written consent and stayed close to, but behind, their baby during the experiment.

## Supporting Information

Video S1 Biological and non-biological motion along an elliptical path (3 s, low resolution animation, 25 fps)
(15.12 MB AVI)

Video S2 Biological and non-biological motion along a circular path (3 s, low resolution animation, 25 fps)
(15.12 MB AVI)
